# Effects of marine biotoxins on drug-metabolizing cytochrome P450 enzymes and their regulation in mammalian cells

**DOI:** 10.1007/s00204-024-03694-6

**Published:** 2024-02-28

**Authors:** Leonie T. D. Würger, Jimmy Alarcan, Albert Braeuning

**Affiliations:** Department Food Safety, German Federal Institute for Risk Assessment, Max-Dohrn-Str. 8-10, 10589 Berlin, Germany

**Keywords:** Drug metabolism, Okadaic acid, Liver, CYP, Nuclear receptors

## Abstract

Marine biotoxins are a heterogenous group of natural toxins, which are able to trigger different types of toxicological responses in animals and humans. Health effects arising from exposure to marine biotoxins are ranging, for example, from gastrointestinal symptoms to neurological effects, depending on the individual toxin(s) ingested. Recent research has shown that the marine biotoxin okadaic acid (OA) can strongly diminish the expression of drug-metabolizing cytochrome P450 (CYP) enzymes in human liver cells by a mechanism involving proinflammatory signaling. By doing so, OA may interfere with the metabolic barrier function of liver and intestine, and thus alter the toxico- or pharmacokinetic properties of other compounds. Such effects of marine biotoxins on drug and xenobiotic metabolism have, however, not been much in the focus of research yet. In this review, we present the current knowledge on the effects of marine biotoxins on CYP enzymes in mammalian cells. In addition, the role of CYP-regulating nuclear receptors as well as inflammatory signaling in the regulation of CYPs by marine biotoxins is discussed. Strong evidence is available for effects of OA on CYP enzymes, along with information about possible molecular mechanisms. For other marine biotoxins, knowledge on effects on drug metabolism, however, is scarce.

## Introduction

Marine biotoxins are a heterogeneous group of natural compounds, which are produced by marine microorganisms and can exert toxic effects in humans and animals, when for example ingested by consumers via contaminated shellfish. Marine biotoxins can be classified into different groups according to the most prominent adverse effects they induce, such as diarrhetic shellfish poisoning (DSP) toxins (caused by azaspiracids (AZA), dinophysistoxins (DTX), pectenotoxins (PTX), and yessotoxin (YTX)), amnesic shellfish poisoning toxins (domoic acid), paralytic shellfish poisoning toxins (saxitoxins), and neurotoxic shellfish poisoning toxins (brevetoxins (PbTX)). For details on marine biotoxin classes, chemistry, occurrence and related adverse health effects, please refer to (Gerssen et al. 2010; Kalaitzis et al. 2010; Morabito et al. 2018; Visciano et al. 2016). The molecular effects causing the different types of toxicity are still not fully understood for many of these compounds.

Okadaic acid (OA) is a dinophysis toxin and the most prominent example of a DSP toxin (Fu et al. 2019; Valdiglesias et al. 2013). It has been shown in animal experiments as well as in vitro that OA-induced DSP is linked to a destruction of the integrity of the intestinal epithelium barrier (Dietrich et al. 2019; Huang et al. 2023; Vale and Botana 2008). This barrier is formed by a layer of enterocytes, which are connected by strong tight junctions in order to prevent a paracellular passage of compounds. Besides the physical barrier function of the intestinal epithelium, intestinal cells and parenchymal liver cells, the hepatocytes, form a biochemical and metabolic barrier against orally ingested foreign compounds, by expressing high levels of drug- and xenobiotic-metabolizing enzymes, as well as transporters responsible for the excretion of xenobiotics and their metabolites. Among the enzymes involved in drug metabolism, cytochrome P450 (CYP) enzymes constitute the most important group.

Integrity of the metabolic barrier can be of high relevance, as alterations in hepatic and intestinal CYP expression and activity caused by foreign compounds are a frequent cause for interindividual variations in drug susceptibility (Zanger and Schwab 2013), as well as for drug-drug interactions (Almond et al. 2009; Galetin et al. 2010; Nettleton and Einolf 2011). Moreover, interference of foreign compounds with CYP activities may also be the underlying cause of non-additive mixture effects of different toxic compounds (Braeuning et al. 2022; Braeuning and Marx-Stoelting 2021; Lasch et al. 2021).

Besides documented effects on the physical intestinal barrier, it has recently been demonstrated that OA affects the expression of various CYP isoform relevant for drug and xenobiotic metabolism in human HepaRG liver cells in vitro (Wuerger et al. 2022, 2023), suggesting that also in vivo the biochemical barrier function of the organ is likely to be affected. This aspect of toxicity has not yet been much in the focus of research on marine biotoxins. Here, we were therefore present and review the current knowledge on the effects of marine biotoxins on CYP enzymes and CYP-regulating nuclear receptors in mammalian species. It should be noted here that marine biotoxins can also be metabolized by CYP enzymes, as for example documented by (Alarcan et al. 2017; Kolrep et al. 2016, 2017). Thus, effects of marine biotoxins on CYP activity or regulation might in turn affect the metabolism and thus the toxicity of marine biotoxins. Nonetheless, this aspect is not in the focus of this review article, which is centered around the effect of marine biotoxins of CYP expression, activity and regulation.

## Literature search strategy

Therefore, searches on the NCBI (National Center for Biotechnology Information) PubMed database (https://pubmed.ncbi.nlm.nih.gov/) were performed to identify relevant publications dealing with effects of marine biotoxins on CYP expression, induction, and activity. The database was searched for different classes of marine biotoxins, not limited to DSP toxins: dinophysis toxins (incl. OA), pectenotoxins, spirolides, yessotoxins, azaspiracides, brevetoxins, and ciguatoxins (with both, group names as well as individual toxin names, and their commonly used abbreviations, as search terms). Search for marine biotoxins was combined with a search for a mention of CYP enzymes or relevant CYP-regulating xeno-sensing receptors (i.e., mainly the aryl hydrocarbon receptor (AHR), the constitutive androstane receptor (CAR), and the pregnane-X-receptor (PXR)), again used as search terms in full or abbreviated.

### Overview of literature search results

**Fig. 1:**
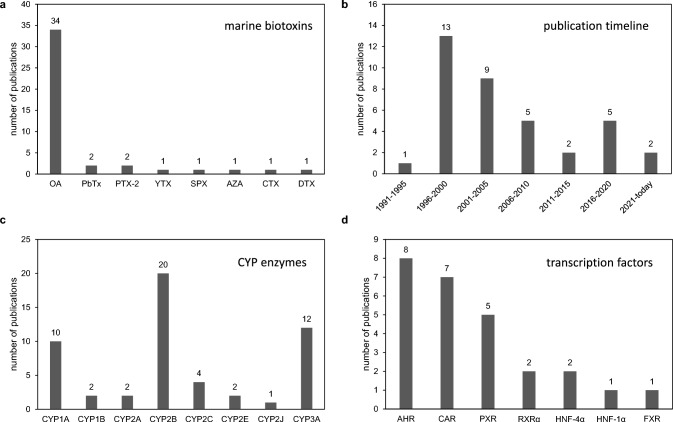
Results of a literature search on the effects of marine biotoxins on the regulation of CYP family 1–3 enzymes in mammalian cells. **a** Numbers of publications containing data on effects of individual marine biotoxins on CYP enzymes. **b** Illustration of publication timeline of relevant papers. **c** Numbers of papers in which the regulation of specific CYP subfamilies has been studied under the influence of marine biotoxins. **d** Identity of the different xeno-sensors/nuclear receptors, which have been studied in the context of CYP regulation by marine biotoxins

Search until May 16, 2023, yielded a total of 37 relevant publications after removal of non-relevant hits, for example papers describing the metabolism of marine biotoxins by CYPs, but not the regulation of CYPs by marine biotoxins. Moreover, results were filtered for mammalian CYPs from CYP subfamilies 1–3, to focus on mammalian drug and xenobiotic metabolism. Papers dealing with other species or CYP families were therefore also excluded. Given the fact that the numbers of publications retrieved with PubMed searches for many research topics are very often three- or four-digit, this gave a first indication that the field had not been subject of extensive research in the past. The first striking observation was that okadaic acid was the toxin of choice in the vast majority of papers (34 out of 37 publications). By contrast, all other toxins, namely AZA1-3, PTX2, DTX1-2, YTX, SPX (spirolide), CTX (ciguatoxin), and PbTX, had been subject of research in only in one or two papers. This is visualized in Fig. 1A. Please note that more than one toxin has been used in several studies. Therefore, the total numbers in the diagram (and subsequent diagrams) may exceed the overall number of papers used as a data source. A publication peak was noted between 1996 and 2005, while the numbers of papers published regarding the regulation of CYPs by marine biotoxins declined somewhat afterwards, with an average of not more than one publication per year or even less since 2011 (Fig. 1B). The CYP subfamily mentioned most in the published literature as being regulated by marine biotoxins (i.e., mainly be okadaic acid), was CYP2B (20 publications), followed by CYP1A (10 publications), CYP3A (12 publications), and CYP2C (4 publications). Other CYP subfamilies were studied less often (Fig. 1C). Among the xenobiotic-sensing receptors, research was rather similarly distributed over the classic xeno-sensors AHR (8 publications), CAR (7 publications), and PXR (5 publications), while some research has also been performed on the nuclear receptors retinoid-X-receptor alpha (RXRα), farnesoid-X-receptor (FXR), and the different hepatocyte nuclear factors (HNFs) (Fig. 1D).

**Fig. 2:**
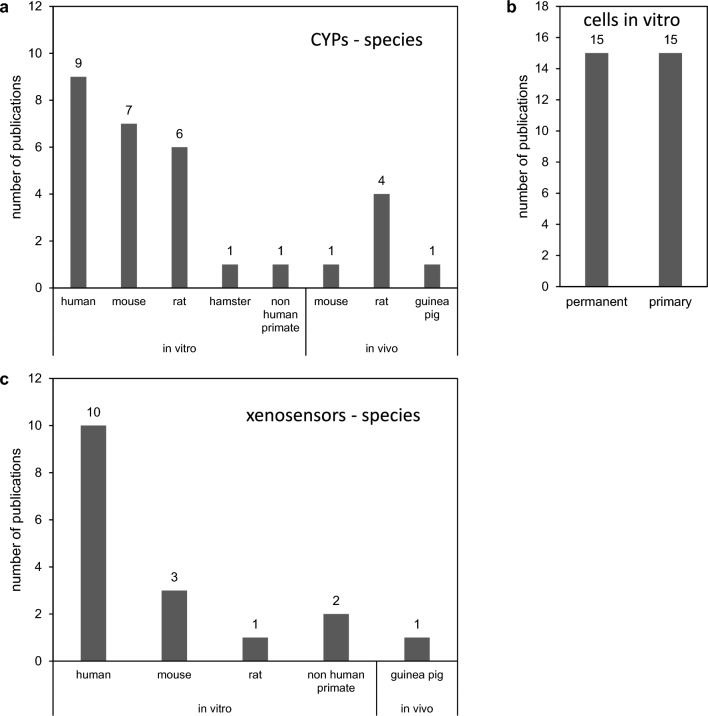
Overview of species and in vitro/in vivo model systems in research on CYP regulation by marine biotoxins. **a** Species origin of model systems in vitro and in vivo for research on CYP regulation by marine biotoxins. **b** Use of primary or permanent cell lines for in vitro research on marine biotoxin effects of CYP regulation. **c** Species origin of model systems in vitro and in vivo for research on the regulation of CYP-regulating xeno-sensors by marine biotoxins

Most of the work on CYP regulation by marine biotoxins has been done in vitro. To this end, human cells have been used most frequently, followed by cells of mouse and rat origin (Fig. 2A). Research in vitro was equally distributed between the use of primary cells and permanent cell lines (Fig. 2B). Studies involving in vivo research were rarer (6 publications), and were done in rats or mice or (Fig. 2A). With respect to nuclear receptor involvement, a clear preference of researchers for human cells and receptors (10 publications) was evident, whereas nuclear receptors from other species were studied only occasionally (Fig. 2C).

### Regulation of CYP family 1 members by marine biotoxins

CYPs from family 1 are known to metabolize primarily planar and hydrophobic substrates. Accordingly, the AHR, activated for example by dioxins and polycyclic aromatic hydrocarbons, is a major transcriptional regulator of the expression of CYP family 1 members (Abel and Haarmann-Stemmann 2010; Haarmann-Stemmann and Abel 2006). CYP 1 regulation by marine biotoxins was investigated in 10 different papers (Alarcan et al. 2017; Ferron et al. 2016; Hukkanen et al. 2000; Oesch-Bartlomowicz et al. 1997; Posti et al. 1999; Shimoyama et al. 2014; Sidhu and Omiecinski 1997; Tamaki et al. 2005; Wuerger et al. 2022; Wuerger et al. 2023), with a total of 21 different entries for individual CYPs, experimental systems and toxins. A summary of the observations along with the respective references is provided in Table 1. OA was most intensively investigated, while PTX-2 was used in two studies. Analyses of effects of other marine biotoxins on CYP1A regulation have not been published. Data for CYP1A1 regulation by OA almost consistently point towards a downregulation of CYP1A1 in different human and rodent in vitro systems (Table 1). No effect of OA on CYP1A1 has been observed in a single paper, where the toxin did not affect the TCDD-induced levels of CYP1A1 in human A549 lung carcinoma cells (Hukkanen et al. 2000). The remaining studies listed in Table 1 are dealing with hepatic cells from rodents or humans; and based on the available data, a general consistent downregulation of CYP1A1 in human and rodent liver cells by OA can be concluded. This appears to apply to both, basal CYP1A1 expression, as well as CYP1A1 expression activated by ligands of the AHR (Table 1). In one study, the effect of OA on TCDD-induced CYP1A1 levels has additionally been investigated in human intestinal and breast cells, and a downregulation of CYP1A1, similar to what was seen in liver cells, was observed here (Shimoyama et al. 2014). Similar observations were made for OA-dependent regulation of CYP1A2, where also a downregulation of the CYP isoform by OA was observed, both in its basal and AHR activator-induced state (Table 1). CYP1B1 expression alterations by OA have been investigated in only two studies with human in vitro models, where a slight but not statistically significant upregulation of TCDD-induced CYP1B1 levels in A549 cells was observed in one publication (Hukkanen et al. 2000), whereas downregulation was observed in liver cells by others (Shimoyama et al. 2014). In contrast to the CYP-repression effects of OA, the few data available for CYP1 regulation by PTX-2 show consistent upregulation of CYPs 1A1 and 1A2 in human cells by this toxin (Table 1).

**Table 1:** Effects of marine biotoxins on CYP1 isoforms

CYP1	Species	In vivo/in vitro	Toxin	Regulation	Inducer	Source
1A	Human	In vitro	PTX-2	–	No	Alarcan (2017)
1A1	Human	In vitro	PTX-2	↑	No	Alarcan (2017)
1A1	Human	In vitro	PTX-2	↑	No	Alarcan (2017)
1A1	Human	In vitro	OA	–	Yes	Hukkanen (2000)
1A1	Rat	In vitro	OA	–	No	Oesch-Bartlomiwicz (1997)
1A1	Rat	In vitro	OA	↓	Yes	Posti (1999)
1A1	Human	In vitro	OA	↓	Yes	Shimoyama (2014)
1A1	Rat	In vitro	OA	↓	Yes	Sidhu (1997)
1A1	Mouse	In vitro	OA	↓	No	Tamaki (2005)
1A1	Human	In vitro	OA	↓	Yes/no	Wuerger (2022)
1A1	Human	In vitro	OA	↓	No	Wuerger (2023)
1A1/2	Rat	In vitro	OA	–	No	Oesch-Bartlomiwicz (1997)
1A2	Human	In vitro	PTX-2	↑	No	Alarcan (2017)
1A2	Human	In vitro	PTX-2	↑	No	Alarcan (2017)
1A2	Human	In vitro	OA	↓	No	Ferron (2016)
1A2	Human	In vitro	PTX-2	↑	No	Ferron (2016)
1A2	Rat	In vitro	OA	↓	Yes	Posti (1999)
1A2	Mouse	In vitro	OA	↓	No	Tamaki (2005)
1A2	Human	In vitro	OA	↓	Yes/no	Wuerger (2022)
1B1	Human	In vitro	OA	↑	Yes	Hukkanen (2000)
1B1	Human	In vitro	OA	↓	Yes	Shimoyama (2014)

Effects of marine biotoxins on CYP family 1 members might, in principle, be indirectly caused by interference with the xeno-sensor and key CYP1A/CYP1B regulator AHR. No or only minor effects of OA on AHR expression or activity were observed in some studies with different models (Alarcan et al. 2017; Kurl 1994; Shimoyama et al. 2014); or downregulation of AHR expression in human cells occurred only at OA concentrations much higher than needed for CYP1A1/1A2 repression (Wuerger et al. 2022). This may indicate that a mechanism distinct from simple regulation of the overall cellular amount of the AHR is responsible for the OA-mediated decrease in CYP1A. On the other hand, increased nuclear AHR localization has been observed in a human epidermis cell line after treatment with OA (Ikuta et al. 2004). Moreover, it was observed in Hepa-1 mouse liver cells that OA does not affect AHR DNA binding and basal AHR-dependent transcription, but increased TCDD-dependent transcription mediated by AHR (Li and Dougherty 1997). OA also appears to be able to affect phosphorylation of the AHR dimerization partner ARNT in COS-1 monkey kidney cells (Levine and Perdew 2002). It is, however, not yet clear, whether these observations result in consequences for the AHR/ARNT dimer and its transactivation potential. Shimoyama and co-workers have conducted mechanistic analyses of AHR activation in human cells and discovered an AHR-independent pathway involving the phosphorylation and dephosphorylation of the transcription factor Sp1 at Ser-59 by protein phosphatase 2A (PP2A), thus offering a possible molecular explanation of the inhibitory effect of OA on CYP1A1 via PP2A inhibition and interference with SP1 phosphorylation (Shimoyama et al. 2014).

### Regulation of CYP family 2 members by marine biotoxins

The CYP2 family gathers many isoforms and metabolizes a wide range of drugs, as for example reviewed by (Pelkonen et al. 2008): Key enzymes from this family are CYP2A6, which typically metabolizes smaller planar molecules (e.g., nicotine), CYP2B6, which catalyzes the biotransformation of neutral molecules, CYP2D6 being well known for its important role in drug metabolism and its genetic polymorphisms, or CYP2E1, well-expressed in the liver and mainly involved in the metabolism of small molecules including ethanol. Members of the CYP2C (e.g., CYP2C9, 2C8, and 2C19) metabolize a large number of commonly used drugs such as fluoxetine, fluvastatin, diclofenac, diazepam, mephenytoin or omeprazole. CYP2 regulation by marine biotoxins was investigated in 23 different papers (Abe et al. 2017; Alarcan et al. 2017; Ferron et al. 2016; Gahrs et al. 2013; Ganem et al. 1999; Honkakoski and Negishi 1998; Inoue et al. 2006; Joannard et al. 2000; Kawamoto et al. 1999; Kawamura et al. 1999; Morey et al. 2008; Nirodi et al. 1996; Posti et al. 1999; Pustylnyak et al. 2005; Samudre et al. 2002; Sidhu and Omiecinski 1997; Swales et al. 2005; Tohkin et al. 1996; Wuerger et al. 2022, 2023; Yamasaki et al. 2018; Yoshinari et al. 2003; Zhang et al. 2006), as summarized in Table 2. Thus, CYP2 family members constitute the CYP enzymes which have been most often mentioned in the context of CYP regulation by marine biotoxins. Notably, 6 of those studies are vivo trials, mostly in rats. Similar to studies of the CYP1 family, OA was the most investigated toxin, while only few studies included on PTX-2 or CTX. In short, following a single dose administration of CTX to male C57/BL6 mice, gene expression analysis in the liver revealed upregulation of CYPs 2B9, 2B10, 2B13, 2E1, and 2J9, while CYPs 2J11 and 2J13 were downregulated (Morey et al. 2008). Data for PTX-2 showed an upregulation of CYP2B6, 2C9, and 2C19 following 24 h treatment in human HepaRG liver cells (Alarcan et al. 2017). These findings at the gene expression level were correlated with increases in the enzymatic activity of 2C9 and 2C19 following 72 h incubation in HepaRG cells (Ferron et al. 2016). Contrary to the upregulations observed for CTX and PTX-2, data in regards to CYP modulation by OA mostly points towards a downregulation of CYP2 enzymes (see Table 2). It is important to note that in most studies, OA was used as pre-treatment before incubation with the indirect CAR activator phenobarbital. Examples of CYP downregulation include CYPs 2B1, 2B2, and 2B10, observed both in in vitro studies using mouse or rat cells. These findings were also reported in most of the in vivo studies conducted in rat (4 out of 5). Data in human liver HepaRG cells show not only gene expression downregulation for *CYP2C8*, *CYP2C9*, *CYP2C19*, and *CYP2E1*, but also decreased CYP activity (Wuerger et al. 2022). In addition, decreases in the activity of CYP2C9 and CYP2C19 following 72 h incubation in HepaRG cells were observed by (Ferron et al. 2016). Conflicting data were reported for CYPB26: while (Wuerger et al. 2022) observed gene downregulation, upregulation was observed by (Swales et al. 2005) (mRNA) and (Inoue et al. 2006) (reporter assay). However, it is important to note that the reported upregulation occurred at most when OA was co-incubated with the CAR activator 1,4-bis [2-(3,5-dichloropyridyloxy)] benzene (TCPOBOP). This outcome points towards the key importance of CAR. Indeed, CYP 2 family enzymes, and especially CYP2B6, are under the transcriptional regulation of CAR. Under physiological conditions, CAR is located in the cytoplasm in an inactive state due to a multi-protein retention complex constituted of heat-shock protein (HSP) 90 and CAR cytoplasmic retention protein (CCRP). HSP70 has also been shown to stabilize this complex in the inactive state (Timsit and Negishi 2014; Yoshinari et al. 2003).

**Table 2:** Effects of marine biotoxins on CYP2 isoforms

CYP2	Species	In vivo/in vitro	Toxin	Regulation	Inducer	Source
2A	Hamster	In vitro	OA	↑	Yes	Tohkin (1996)
2A5	Rat	In vitro	OA	↓	Yes	Posti (1999)
2A8	Hamster	In vitro	OA	↑	Yes	Tohkin (1996)
2B	Rat	In vivo	OA	↓	Yes	Joannard (2000)
2B	Rat	In vivo	OA	↓	Yes	Pustylnyak (2005)
2B	Rat	In vivo	OA	↓	Yes	Samudre (2002)
2B1	Rat	In vitro	OA	↓	Yes	Gahrs (2013)
2B1	Mouse	In vitro	OA	↓	Yes	Ganem (1999)
2B1	Rat	In vitro	OA	↓	Yes	Kawamura (1999)
2B1	Rat	In vitro	OA	↓	Yes	Sidhu (1997)
2B1	Rat	In vivo	OA	↓	Yes	Zhang (2006)
2B1/2	Rat	In vivo	OA	↑	Yes	Nirodi (1996)
2B10	Mouse	In vitro	OA	↓	Yes	Abe (2017)
2B10	Mouse	In vitro	OA	↓	Yes	Honkakoski (1998)
2B10	Mouse	In vitro	OA	↓	Yes	Kawamoto (1999)
2B10	Mice	In vivo	CTX	↑	No	Morey (2008)
2B10	Rat	In vitro	OA	↓	Yes	Posti (1999)
2B10	Mouse	In vitro	OA	↓	Yes	Yoshinari (2003)
2B13	Mouse	In vivo	CTX	↑	No	Morey (2008)
2B13	Mouse	In vivo	CTX	↑	No	Morey (2008)
2B2	Mouse	In vitro	OA	↓	Yes	Ganem (1999)
2B2	Rat	In vivo	OA	↓	Yes	Joannard (2000)
2B2	Rat	In vitro	OA	↓	Yes	Kawamura (1999)
2B2	Rat	In vivo	OA	↑	Yes	Nirodi (1996)
2B2	Rat	In vitro	OA	↓	Yes	Sidhu (1997)
2B2	Rat	In vivo	OA	↓	Yes	Zhang (2006)
2B6	Human	In vitro	PTX-2	↑	No	Alarcan (2017)
2B6	Human	In vitro	OA	↑	Yes	Inoue (2006)
2B6	Human	In vitro	OA	↑	Yes	Swales (2005)
2B6	Human	In vitro	OA	↓	Yes/no	Wuerger (2022)
2B6	Human	In vitro	OA	↓	No	Wuerger (2023)
2B9	Mouse	In vivo	CTX	↑	No	Morey (2008)
2C19	Human	In vitro	PTX-2	↑	No	Alarcan (2017)
2C19	Human	In vitro	OA	↓	No	Ferron (2016)
2C19	Human	In vitro	PTX-2	↑	No	Ferron (2016)
2C19	Human	In vitro	OA	↓	Yes/no	Wuerger (2022)
2C55	Mouse	In vitro	OA	↓	Yes	Yamasaki (2018)
2C8	Human	In vitro	OA	↓	Yes/no	Wuerger (2022)
2C9	Human	In vitro	PTX-2	↑	No	Alarcan (2017)
2C9	Human	In vitro	OA	↓	No	Ferron (2016)
2C9	Human	In vitro	PTX-2	↑	No	Ferron (2016)
2C9	Human	In vitro	OA	↓	Yes/no	Wuerger (2022)
2E1	Mouse	In vivo	CTX	↑	No	Morey (2008)
2E1	Human	In vitro	OA	↓	Yes/no	Wuerger (2022)
2J11	Mouse	In vivo	CTX	↓	No	Morey (2008)
2J13	Mouse	In vivo	CTX	↓	No	Morey (2008)
2J9	Mouse	In vivo	CTX	↑	No	Morey (2008)

### Regulation of CYP family 3 members by marine biotoxins

CYP3 family members engaged in xenobiotic metabolism in humans or rodents are the different isoforms of sub-family CYP3A, which is mainly regulated by PXR; for example, see the review by (Tompkins and Wallace 2007). Key isoforms in humans include CYP3A4, CYP3A5 and CYP3A7 (Pelkonen et al. 2008). CYP3A regulation by marine biotoxins has been studied in 12 different publications (Alarcan et al. 2017; Ding and Staudinger 2005; Ferron et al. 2016; Gahrs et al. 2013; Joannard et al. 2000; Morey et al. 2008; Swales et al. 2005; Wuerger et al. 2022, 2023; Yamasaki et al. 2018; Yokobori et al. 2019; Zhang et al. 2006), and again OA was the main biotoxin studied, with PTX-2 being investigated as a second toxin (Table 3). Data univocally show a downregulation of CYP3A enzymes, in cell line models of mostly human, more rarely of rodent origin, as well as in rodent in vivo studies (Table 3). Moreover, results also show decreased CYP3A4 activity following 24 h (Wuerger et al. 2022) or 72 h incubation in HepaRG cells (Ferron et al. 2016). Administration of CTX to male C57/BL6 mice led to CYP3A44 downregulation in the liver (Morey et al. 2008). In contrast to the generally rather CYP-repressive effects of OA, available data show no effect or very slight upregulation of CYPs 3A4 and 3A5 by PTX-2 in human liver cells (Table 1). In a HepG2-hPXR luciferase reporter assay, OA induced luciferase activity, suggesting a PXR-dependent activation of the luciferase reporter construct through the XREM sequence of the human *CYP3A4* promoter (Ferron et al. 2016). Conversely, in transfected HEK-T cells, OA provoked strong decreases in firefly luciferase signals in human PXR and RXRα transactivation assays (Wuerger et al. 2023).

**Table 3:** Effects of marine biotoxins on CYP3 isoforms

CYP3	Species	In vivo/in vitro	Toxin	Regulation	Inducer	Source
3A	Rat	In vivo	OA	↓	Yes	Joannard (2000)
3A	Rat	In vivo	OA	↓	Yes	Zhang (2006)
3A1	Rat	In vitro	OA	↓	Yes	Gahrs (2013)
3A11	Non-human primate	In vitro	OA	↓	Yes	Ding (2005)
3A11	Mouse	In vitro	OA	↓	Yes	Yamasaki (2018)
3A4	Human	In vitro	PTX-2	↑	No	Alarcan (2017)
3A4	Human	In vitro	OA	↓	No	Ferron (2016)
3A4	Human	In vitro	PTX-2	↑	No	Ferron (2016)
3A4	Human	In vitro	OA	↓	Yes/no	Wuerger (2022)
3A4	Human	In vitro	OA	↓	No	Wuerger (2023)
3A4	Human	In vitro	OA	↓	Yes	Yokobori (2019)
3A44	Mouse	In vivo	CTX	↓	No	Morey (2008)
3A5	Human	In vitro	PTX-2	↑	No	Alarcan (2017)
3A7	Human	In vitro	OA	↓	Yes	Swales (2005)

### Mechanism of action underlying the CYP inhibition by marine biotoxins

The overall inhibitory effects of OA on different CYPs could be in theory explained by a direct interference of OA with the respective xeno-sensing receptor (i.e., AHR, CAR, and PXR). Considering the body of available data, it appears, however, quite unlikely that OA is a ligand for all the different xeno-sensors with their different ligand preferences. In contrast, the inhibitory property of OA towards PP2A may play a role in the constitution of nuclear receptor-protein co-factor complexes through alterations in the phosphorylation status of one or more of these proteins: Phosphorylation-mediated signal regulation in the nuclear receptor context has gained importance since the discovery of indirect CAR activation by the anti-epileptic drug phenobarbital (PB). In brief, it has been shown that CAR is being inactivated by phosphorylation at Thr-38 residue, while the dephosphorylation is activating the receptor (Negishi et al. 2020). These processes are physiologically regulated by cell growth signals, but PB can disrupt these cell signals to induce dephosphorylation, thus activating CAR indirectly. Another important step is the PP2A-mediated dephosphorylation of CAR, which is needed for nuclear translocation and heterodimerization with RXRα. The involvement of PP2A in the regulation of the AHR has also been demonstrated in several studies. For instance, the dephosphorylation of the transcription factor SP1 at serine 59 by PP2A was required for induction of *CYP1A1* transcription in response to 2,3,7,8-tetrachlorodibenzo-*p*-dioxin or omeprazole in human cells (Shimoyama et al. 2014). Another team showed that specific PP2A B56α complexes participated in AHR-mediated induction of *Cyp1a1* in mice (Chen et al. 2022). In light of these aspects, the inhibitory property of OA towards PP2A very likely plays a role in the repression of AHR, CAR, and possibly also PXR target genes.

In addition to rather direct effects of PP2A on nuclear receptors and their co-factors, crosstalk with other signaling pathways, directly or indirectly affected by OA, might be responsible for the observed effects. Reviewing all possible protein–protein interactions and signaling crosstalk mechanism is beyond the scope of this section. The following section will therefore be focused on the role of pro-inflammatory signaling.

### Inflammation as mediator of CYP inhibition by marine biotoxins

OA leads to severe downregulation of CYP enzymes in human liver cells, while the levels of proinflammatory cytokines simultaneously rise (Wuerger et al. 2022, 2023). It is known that elevated levels of proinflammatory cytokines lead to the downregulation of several different CYP enzymes in human liver cells; for example, see (Keller et al. 2016; Kugler et al. 2020; Tanner et al. 2018; Zanger and Schwab 2013). Moreover, recent data demonstrate that proinflammatory signaling is causative for CYP downregulation by OA in human HepaRG cells (Wuerger et al. 2023). It has been shown that the p65 subunit of NF-κB disrupts DNA binding of the PXR/RXRα complex on the human *CYP3A4* gene promoter region (Gu et al. 2006). Additionally, interleukin 6 (IL-6) was shown to specifically inhibit rifampicin- and PB-mediated induction of the human *CYP2B6*, *CYP2C8*/*9*, and *CYP3A4* genes (Pascussi et al. 2000). Therefore, a closer look on the proinflammatory potential of other marine biotoxins is of interest.

Brevetoxins (PbTx) have shown to induce proinflammatory cytokines like IL-2 and TNFα in mouse-derived MH-S cells, but also elevate the levels of the anti-inflammatory cytokine IL-4 (Sas and Baatz 2010). Furthermore, Hilderbrand et al. showed elevated levels of IL-6 in mouse bone marrow-derived mast cells after exposure to PbTx (Hilderbrand et al. 2011). However, McCall and co-workers recently found no significant effect on IL-1β, IL-6 and TNF-α in human THP-1 cells, but elevated levels of anti-inflammatory IL-4 (McCall et al. 2022).

The amnesic shellfish poisoning marine biotoxin domoic acid also leads to expression of proinflammatory cytokines, like IL-1β and TNFα, in the hippocampus of mice (Lu et al. 2013; Ryan et al. 2005). However, Ryan et al. also showed an inhibition of NF-κB activation in mice (Ryan et al. 2005). Furthermore, there was no detectable increase in interleukin levels in the blood of non-human primates after exposure to domoic acid, which argues against the possibility of a systemic inflammatory response in humans (Petroff et al. 2022).

CTX can induce the chronic inflammatory response syndrome (CIRS). In patients with CIRS, several inflammatory markers are upregulated in their blood (Ryan et al. 2015). This leads to the assumption that CTXs are strongly upregulating proinflammatory cytokines. However, differences between the different CTXs have been observed. The highly potent toxin P-CTX-1B was able to strongly upregulate the expression of IL-1β, TNFα and IL-6 in murine RAW 264.7 cells, a murine macrophage cell line, but also upregulated the anti-inflammatory IL-10. P-CTX-3C, however, did not show such a strong proinflammatory potential (Matsui et al. 2010). P-CTX-1 even showed a downregulation of the proinflammatory marker IL-1β in mouse brain, liver and blood (Ryan et al. 2010). This suggests that the proinflammatory potential highly depends on the specific CTX tested.

In the group of the STX, only neosaxitoxin (NeoSTX) has been researched regarding its inflammatory potential so far. NeoSTX is able to directly counteract the effects of OA regarding the release of proinflammatory cytokines, as it was able to block the release of TNFα into the blood stream in horses induced by OA (Montero et al. 2021). Furthermore, NeoSTX was able to inhibit the expression of proinflammatory factors IL-1β, TNFα and iNOS in RAW 264.7 cells (Montero et al. 2020). Based on these results, it can be assumed that the effect of NeoSTX on the inflammation is directly opposing the effect of OA.

The inflammatory potential of YTX, PTX and AZA have only been examined in one publication so far. YTX and AZA1 were able to translocate NF-κB, similar to OA, in a rat enteric glial cell line, which implies a proinflammatory response, while PTX2 showed no effect (Reale et al. 2019).

As evident from the cited sources, there are some marine biotoxins, apart from OA, that show the potential for inducing proinflammatory effects. As cytokine induction has proven to play a role in OA-mediated downregulation of CYP expression in human cells, similar effects might be expected also with other marine biotoxins which provoke a cytokine response in exposed cells. However, not much data is available so far and more extensive evaluation of the toxicological profiles of the different toxins are needed to elucidate further on their proinflammatory potential and thereby on their possible effect on CYP enzymes.

## Summary and conclusion

In summary, the data demonstrate that there has not been much systematic research on the effects marine biotoxins on CYP enzymes, their regulating nuclear receptors, or proinflammatory signaling cascades. The only exception is OA, for which numerous data sets are available. Provided the fact that there is evidence that other marine biotoxins might also interfere with inflammatory signaling, it will of interest for future research to analyze whether the biochemical barrier of the intestine and liver might also be affected by marine biotoxins other than OA. Moreover, mechanistic in vitro studies will enhance our understanding of molecular toxicity of marine biotoxins, and studies of marine biotoxin mixture toxicity will be crucial to predict possible in vivo mixture effects of real-life exposure.
